# A Google Earth-based surveillance system for schistosomiasis japonica implemented in the lower reaches of the Yangtze River, China

**DOI:** 10.1186/1756-3305-4-223

**Published:** 2011-11-27

**Authors:** Le-Ping Sun, You-Sheng Liang, Hong-Hui Wu, Zeng-Xi Tian, Jian-Rong Dai, Kun Yang, Qing-Biao Hong, Xiao-Nong Zhou, Guo-Jing Yang

**Affiliations:** 1 Jiangsu Institute of Parasitic Diseases, Wuxi, Jiangsu, 214064, Key laboratory of the Ministry of Health, China; 2 Office of Leading Group for Schistosomiasis and Other Endemic Diseases Control of Jiangsu Province, Jiangsu Provincial Department of Health, Nanjing, Jiangsu, China; 3 National Institute of Parasitic Diseases, Chinese Center for Disease Control and Prevention, Shanghai, China; 4 School of Public Health and Primary Care, the Chinese University of Hong Kong, Satin, Hong Kong

## Abstract

**Background:**

Due to the success of the national schistosomiasis control programme in China, transmission has been sufficiently reduced in many areas to severely limit identification of areas at risk by conventional snail surveys only. In this study, we imported Google Earth technology and a Global Positioning System (GPS) into the monitoring system for schistosomiasis surveillance of the banks of the Yangtze River in Jiangsu Province, China.

**Methods:**

A total of 45 sites were selected and the risk was assessed monthly by water exposure of sentinel mice at these sites from May to September in 2009 and 2010. The results were assembled and broadcast via the Google Earth platform.

**Results:**

The intensity of schistosomiasis transmission showed peaks of risk in June and September of 2009, while there was only one small peak in June in 2010 as the number of detected positive transmission sites dropped dramatically that year thanks to improved mollusciciding. River ports were found to be areas of particular risk, but ferry terminals and other centres of river-related activities were also problematic.

**Conclusions:**

The results confirm that the surveillance system can be rapidly updated and easily maintained, which proves the Google Earth approach to be a user-friendly, inexpensive warning system for schistosomiasis risk.

## Background

The transmission of schistosomiasis depends on the presence of aquatic or amphibious intermediate snail hosts and the disease is therefore closely related to water-bodies and water courses [1,2]. The maturation process in the snail is governed by the ambient temperature, while the shedding of cercariae (the infective stage) from the snail not only requires water of proper temperature but also sufficient day light [3,4]. Once emerged from their snail host, the cercariae move towards the surface, contaminating the water and thus turning the area into one of risk for schistosome infection. When humans and/or other definitive animal hosts are exposed to water under such circumstances, the cercariae home in, penetrate the skin and move through the systemic circulation via the lungs and liver to finally settle in the mesenteric microcirculation [3].

Schistosomiasis japonica, distributed in China and the Philippines, is one of the three major forms of human schistosomiasis which all belong to the neglected tropical diseases (NTDs) [1,5]. The morbidity due to infection by *Schistosoma japonicum *is more severe than that caused by the two other main species infective to humans [6]. The transmission foci of schistosomiasis in China are mainly located in five provinces around the lakes in central China including the middle and lower reaches of the Yangtze River [7-9]. Infected humans and bovines in these areas account for 97.2% and 91.2%, respectively, of the total number of cases in the country [7]. Transmission depends entirely on the distribution of the intermediate snail host *Oncomelania hupensis *and the seasonal risk of infection falls between May and October in alliance with the annual flooding of the Yangtze River [8,10].

Specific schistosomiasis surveillance in China was introduced in the late 1980s, when scientists conducted a series of preliminary studies to investigate the usefulness of monitoring methods in regions where transmission interruption had been achieved [11-13]. In the early 1990s, a national surveillance network of schistosomiasis was formally established in combination with the launch of the World Bank Loan Project (WBLP) on schistosomiasis control in China [14]. Since then, long-term, continuous and systematic monitoring was gradually developed and integrated with the national schistosomiasis control programme. Dynamic transmission data, as well as information on environmental factors, are now collected via this surveillance system, making it possible to annually update the epidemic status of schistosomiasis in China [15,16]. The popularization of computerized network applications, Remote Sensing (RS) and Geographical Information Systems (GIS) technologies towards the end of the 20th century facilitated the introduction of surveillance characterized by rapid assessment and timely feedback [17-19]. GIS and RS have contributed to prediction of the future distribution and transmission of the disease, forecasting the potential impact of global warming on its distribution, and the study of the potential impact of the south-north water transfer project on the transmission of schistosomiasis in China [1,20].

Both human and livestock infection rates in Jiangsu Province along the lower reaches of the Yangtze River have reached the transmission criterion of < 1% prevalence [21,22]. Although a very positive development, these low endemicity levels makes it difficult to identify the exact extent of infection risk by conventional surveillance systems. Rapid evaluation of water-bodies and up-to-date risk information is an absolute requirement if transmission is to be further mitigated. To deal with this situation in practise, we propose a novel warning system based on the exposure of sentinel mice to water in areas under surveillance with the spatial data monitored by GIS combined with a Global Positioning System (GPS) [23] supported by Google Earth software. The overall aim was to explore a new approach to monitoring schistosomiasis japonica in low-endemic areas.

## Methods

The study focused on finding positive sites (areas at risk for both humans and domestic animals) indicated by infected sentinel mice, along with snails and humans surveyed in parallel.

**Figure 1:**
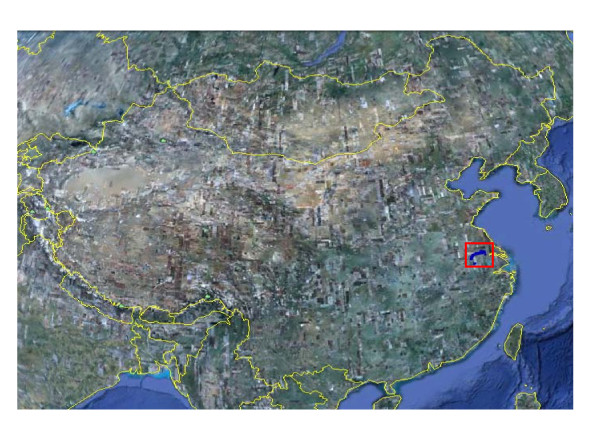
**Surveillance region and sites in Jiangsu province, China**.

**Figure 2:**
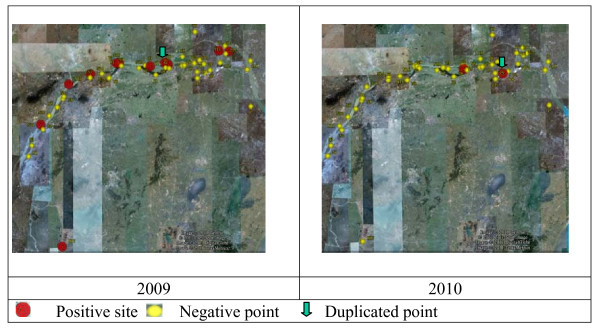
**Google Earth based sentinel mice surveillance results in 2009 and 2010**.

Professional training courses were run prior to the field work at the provincial, municipal and county levels to make sure that a unified plan, a unified approach, a unified time and uniform norms were followed to guarantee accuracy of the results. Local staff controlled the on-site activities (keeping accidental loss of mice as low as possible), while raising and dissections of mice were carried out by a professional team of Jiangsu Institute of Parasitic Diseases (JIPD) at a high-security laboratory, i.e. the Specific Pathogen Free (SPF) laboratory at the JIPD. The surveillance system was set up following a series of steps detailed below aimed at assuring thorough analysis of on-site information and outcomes:

1. Forty-five monitoring sites were selected in Jiangsu Province. The sites were located approximately 10 km from each other along both banks of the lower reaches of the Yangtze River. All were infested with *O. hupensis *snails and frequently approached by livestock and also commonly chosen by humans for various activities. The geographical coordinates of the surveillance sites, provided by GPS instruments (Garmin Map76), is shown in Table 1 and Figures 1 and 2.

**Table 1:** Localization of the surveillance sites studied (see also Figure 3)

Surveillance site (Type and name)	Code*	North latitude	East longitude
Marshland, Jiangning	N1	31°46'44.32″	118°30'19.55″
Marshland, Jiangning	N2	31°54'50.75″	118°35'45.72″
Dock, Jiangning	N3	31°50'00.79″	118°31'32.11″
Marshland, Jianye	N4	32°04'08.91″	118°43'15.58″
Marshland, Jianye	N5	32°01'32.31″	118°41'04.46″
Riverside, Xiaguan	N6	32°04'48.87″	118°43'34.54″
Marshland, Qixia	N7	32°10'26.17″	118°53'25.69″
Marshland, Qixia	N8	32°10'18.90″	119°00'32.60″
Marshland, Qixia	N9	32°14'01.46″	119°05'09.70″
Marshland, Qixia	N10	32°13'19.52″	119°11'47.61″
Marshland, Pokou	N11	31°56'11.92″	118°34'54.32″
Marshland, Pukou	N12	31°58'58.43″	118°38'04.63″
Marshland, Pukou	N13	32°08'25.57″	118°45'17.57″
Bridge islet, Luhe	N14	32°11'27.85″	118°53'32.75″
Marshland, Luhe	N15	32°11'39.41″	118°57'52.25″
Islet, Luhe	N16	32°14'43.50″	119°03'22.34″
Ferry, Gaochun	N17	31°19'26.10″	118°42'58.07″
Marshland, Dantu	Z1	32°11'10.86″	119°18'04.02″
River, Dantu	Z2	32°13'23.10″	119°21'47.72″
Ferry, Dantu	Z3	32°13'42.14″	119°16'00.59″
Ferry, Dantu	Z4	32°12'30.42″	119°38'25.15″
Factory, Dantu	Z5	32°12'03.12″	119°33'35.53″
Ferry, Runzhou	Z7	32°14'05.03″	119°28'47.90″
River, Runzhou	Z8	32°13'10.57″	119°23'06.24″
Bridge, Jingkou	Z9	32°13'58.51″	119°27'57.95″
Marshland, Jingkou	Z10	32°14'41.19″	119°32'30.66″
Watergate, Jingkou	Z11	32°10'08.86″	119°33'40.03″
Marshland, Zhenjiang	Z12	32°14'45.03″	119°42'12.97″
Marshland, Yangzhong	Z13	32°18'04.63″	119°45'33.73″
Marshland, Yangzhong	Z14	32°12'38.69″	119°52'20.28″
Ferry, Yangzhong	Z15	32°17'27.29″	119°44'18.56″
Ferry, Yizheng	Y1	32°15'03.40″	119°04'11.52″
River, Yizheng	Y2	32°14'06.90″	119°14'06.58″
Marshland, Yizheng	Y3	32°14'37.84″	119°04'23.47″
Harbor, Yangzhou	Y4	32°14'40.58″	119°21'35.24″
Marshland, Yangzhou	Y5	32°16'37.28″	119°28'06.15″
Marshland, Hanjiang	Y6	32°14'10.96″	119°22'45.75″
Marshland, Hanjiang	Y7	32°18'15.46″	119°41'47.20″
Marshland, Hanjiang	Y8	32°15'34.18″	119°34'31.35″
Marshland, Hanjiang	Y9	32°24'08.89″	119°32'51.13″
Marshland, Hanjiang	Y10	32°14'26.06″	119°36'07.14″
Marshland, Jiangdu	Y11	32°19'48.06″	119°44'05.91″
Marshland, Changzhou	C1	32°01'31.97″	119°53'32.48″
Marshland, Taizhou	T1	32°15'15.26″	119°53'33.71″

*The letters N, Z, Y, C and T denote the cities Nanjing, Zhenjiang, Yangzhou, Changzhou and Taizhou, respectively.

2. Systematic snail surveys of the beaches at the monitoring sites were carried out in the spring of 2009 and 2010. A square frame measuring 0.11 m^2 ^was put on the ground every 20 meters and all snails limited by this boundary were recorded, collected and brought back to the laboratory, where they were crushed and microscopically examined for infection [24].

3. Sentinel mice of both genders, weighing 23 ± 3 g (females) and 28 ± 3 g (males), were selected. Wired-mesh, 7 × 7 × 65 cm large, rectangular cages were constructed and divided into five cells of equal size. Pieces of foam plastic were tied to the two ends of each cage to make it float on the water surface. At each surveillance site, two cages (each cell containing 2 mice, each cage 10 mice) were anchored 10-20 m apart. The foam plastic pieces of each cage were adjusted to assure that the abdomen and tail of each mouse would be exposed to the water. Each month during the period May to September, the sentinel mice were left in the cages on the water surface from 10:00 A.M. to 2:00 P.M. for two consecutive days.

Sentinel mice survey followed the guideline of "Schistosomiasis Prevention Handbook " published by Ministry of Health, P. R. China [24].

4. After exposure, the mice were collected, indexed and left in the animal department of the laboratory at an ambient temperature of 22-26°C and a relative humidity of 40-70%. They were killed after 35-40 days (to allow maturation of any infecting schistosome), and dissected taking care to inspect the portal and mesenteric veins for worms and the liver for eggs. Mice with eggs in the liver and/or presence of adult worms were recorded as positive for infection.

Out of 9,000 sentinel mice exposed during the 2-year study, a total of 787 mice (8.74%) were lost. This loss was mainly due to cages floating away or mice accidentally drowned or suffocated during feeding. The remaining 8,213 were sacrificed and investigated; 4,370 in 2009 and 3,843 in 2010. The number of positive mice and the worm burdens for each surveillance site and month were calculated and recorded

5. Data management, presentations and electronic dissemination were supported by Google Earth, version 5.0 and the photographic images of the surveillance sites saved in the photo gallery by Picasa, version 3.1 (Google). For each surveillance site, a database was prepared that included information on location (latitude and longitude), snail distribution and the monitoring results for each month. In addition, the sites were entered as 'landmarks' into Google Earth by coordinates using red colour for positives and yellow for negatives.

The site photographs and the data with respect to the sentinel mice for all surveillance sites were imported into the Google Earth platform. The process from dissection of the mice till electronic inclusion of the data in the Google Earth platform was never longer than three working days.

**Figure 3:**
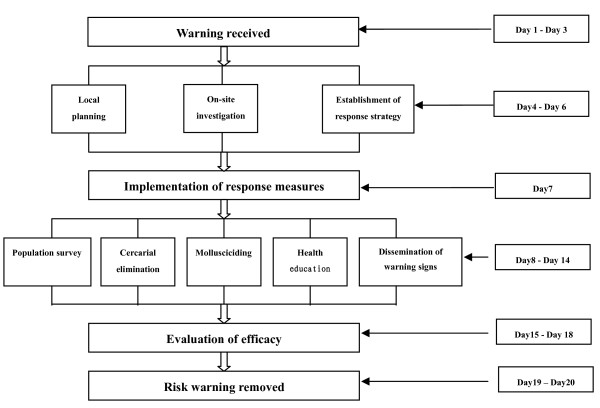
**Flow chart of the surveillance-response system operated via the Google Earth platform**.

6. Information regarding the schistosomiasis infection was regularly sent to the local disease control centre via the Google Earth platform in parallel to being sent to higher levels. A detailed workplan for the local schistosomiasis control was developed and scheduled to be set in motion in case of positive results. It required that professional, technical teams be sent to the sites within 24 hours to survey the environment and establish specific control measures. The plan further required that treatment and protection of high-risk populations be applied within three days and that an evaluation of the response efficacy was prepared within one week (Figure 3).

## Results

**Figure 4:**
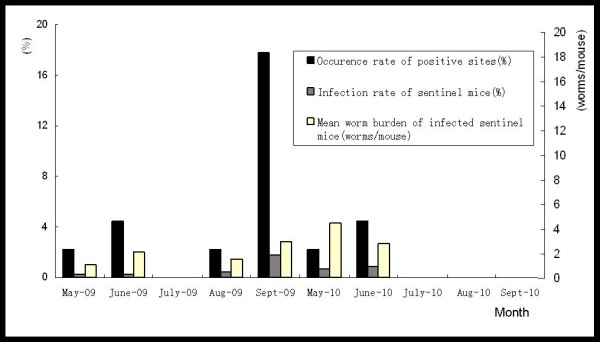
**Surveillance results in different months along Yangtze River in Jiangsu Province**.

The findings are summarized in Table 2, which highlights the large difference between the two years studied. The numbers of positive sites and infected sentinel mice in 2009 were 12 and 23, respectively, which contrasts sharply with the corresponding figures of 3 and 12 in 2010 (Figure 2). The difference is further emphasized by the recorded overall positive rates of sentinel mice in 2009 and 2010 that were 0.53% and 0.31%, respectively. It is worth drawing attention to the fact that the temporal pattern varied between the years with positive findings in the spring of 2009 plus a strong peak in September (particularly reflected by the number of positive sites), while the results in 2010 were limited to positive findings in May and June only (Figure 4). A total of 55 adult *S. japonicum *worms were collected from the 23 positive sentinel mice found in 2009. In spite of finding a much lower number of positive mice in 2010 (12), and only in the beginning of the season, as many as 42 worms were collected from these mice in total.

**Table 2:** Surveillance results in the 45 surveillance sites along the Yangtze River

Time of exposure	Sites positive	Mice dissected	Mice positive	Worms collected	Infection rate (%)	Worm burden*
12-13 May 2009	1	886	2	2	0.23	1.00
10-11 June 2009	2	866	2	4	0.23	2.00
10-11 July 2009	0	866	0	0	0.00	0.00
10-11 Aug 2009	1	886	4	6	0.45	1.50
6-7 Sept 2009	8	866	15	43	1.73	2.87

Subtotal	12	4,370	23	55	0.53	2.39

30-31 May 2010	1	829	6	26	0.72	4.33
18-19 June 2010	2	737	6	16	0.81	2.67
14-15 July 2010	0	795	0	0	0.00	0.00
12-13 Aug 2010	0	652	0	0	0.00	0.00
9-10 Sept 2010	0	830	0	0	0.00	0.00

Subtotal	3	3,843	12	42	0.31	3.50

Total	15	8,213	35	97	0.43	2.77

*Average number of worms per infected mouse

With regard to the surveillance sites, a total of 12 positive ones, including one duplicate record (site found positive at two separate occasions), were detected in 2009, i.e. 24.44% (11/45) or almost a quarter of all sites were at risk that year. There was a small peak in May/June and a strong peak in September mirroring the temporal distribution of infected mice (Figure 4). In 2010 in contrast, only three positive surveillance sites, again including one duplicate were detected over the whole surveillance period, and only in May and June (Table 2).

Positive sites were located at various points of specific human activities: three at river ports, three at ferry terminals and four at fishing centres. Another three positive sites were found at shipyards and at an emerging sand bank in the river, respectively. The highest infection rate was located at the river ports with an infection rate of 2.2% and the highest snail density was detected in a shipyard in 2010. Two of the surveillance-positive points were snail-free (Table 3).

**Table 3:** Surveillance results in relation to type of site

Type of site	Number of sites	Year	**Sites positive**^**a**^	**Snail distribution**^**b**^	**Snail density**^**c**^	**Infection rate (%)**^**d**^	**Worm burden**^**e**^
Shipyard	7	2009	1	0.00	-	0.30 (2/663)	1.00
		
		2010	1	10.00	3.14	1.15 (7/611)	4.00

Ferry terminal	10	2009	3*	3.51	0.49	0.41 (4/969)	1.00
		
		2010	0	-	-	0.00 (0/853)	-

Fishing centre	12	2009	3	44.13	1.47	0.25 (3/1177)	1.67
		
		2010	1	13.00	2.82	0.50 (5/1009)	2.80

New sand bank	3	2009	1	40.29	0.08	0.34 (1/294)	1.00
		
		2010	0	-	-	0.00 (0/263)	-

River port	6	2009	3	32.10	1.86	2.25 (13/578)	3.31
		
		2010	0	-	-	0.00 (0/529)	-

Other	7	2009	0	-	-	0.00 (0/689)	-
		
		2010	0	-	-	0.00 (0/578)	-

Total	45	2009	11	120.03	0.88	0.53 (23/4370)	2.39
		
		2010	2	23.00	2.97	0.31 (12/3843)	3.50

^a^Deemed to be positive based on infection found in sentinel mice. ^b^Estimated size of snail-infested area (hm^2^). ^c^Average number of snails/m^2^. ^d^Number of infected sentinel mice/total number of mice used. ^e^Average number of worms per infected mouse. *No snails found in one of the sites.

The surveillance results were electronically transmitted five times per year (one for each month of the season) to local and superior levels through the Google Earth surveillance system. This was not only done for research and surveillance purposes but also to ensure that emergency measures such as mollusciciding and cercarial elimination were carried out to protect the local population in case of detected risk. The technical staff at the health authorities of the counties (cities, districts) where positive sentinel mice were detected went to the field and screened all high risk populations. A total of 2,968 people were tested serologically by dipstick immunoassay provided by JIPD. Chemotherapy was given to 417 people who tested positively. Niclosamide ethanolamine was applied as cercariacidal to around 25,700 m^2 ^suspected water surfaces and health education was delivered to the people frequenting sites found to be positive. Warning signs were set up along the river banks and protection ointment against schistosome infection was distributed to all considered to be at risk.

In the spring of 2010, mollusciciding was twice applied at the 11 positive sites detected in 2009. In August, 2010, Jiangsu launched a propaganda week of schistosomiasis control in the sensitive regions along the Yangtze River, subjecting a total of 26,421 people of the mobile population working on the river to health examinations with a special focus on schistosomiasis.

## Discussion

The Google Earth and GPS technologies were introduced into the monitoring system for schistosomiasis, in order to 1) visualize the spatiotemporal aspects of the monitoring approach, 2) understand the transmission dynamics of schistosomiasis along the Yangtze River, and 3) provide rapid risk updates for the purpose of immediate emergency responses.

Schistosomiasis japonica impacts human health seriously, hindering social and economic development [25]. After six decades of successful prevention, China has made great progress in schistosomiasis control. The number of infected people has dropped from the initial 11.6 million in 1950s to 0.4 million currently, a decrease of 96.65% [6,26]. Around 80% of all endemic counties (districts, cities) have met the criteria for transmission interruption and control [7,27] and surveillance is rapidly becoming an important part of the national schistosomiasis control programme.

As we arrive at different control stages, the monitoring content, methods and indicators change [28]. While surveillance in the early 1980's was less developed and relatively passive [11-13], an active system based on defined monitoring methods and indicators has now been implemented [15,28]. By the start of the 21st century, 80 fixed surveillance sites are in use for longitudinal monitoring within the endemic areas [9,29]. At the same time, as a supplement to these fixed sites, mobile units have been introduced at the provincial level.

To improve and standardize the approach, and at the same time saving time and money, we integrated the Google Earth software including an operative platform and the use of sentinel mice to establish a modern warning system. Application of this system over two transmission seasons (2009-2010) has proved that this user-friendly approach is inexpensive, yet easily maintained, so lending itself to continuous updating.

This study covered 200 km along the Yangtze River in Jiangsu province and took place between May and September in 2009 and 2010. The overall positive rates of sentinel mice for the two years was 0.4% which is considerably lower than that (26.6%) recorded in 2003 based on 26 surveillance sites in Nanjing, the capital of Jiangsu Province [30]. The results clearly confirm that schistosomiasis transmission has been significantly reduced in Jiangsu province since the implementation of the long-term control plan by the Jiangsu government [21,22].

Two infection peaks were detected in 2009 which is consistent with previous reports [31,32]. This two-peak transmission pattern can be explained by the periodic evolvement of infected snails. The lifespan of the intermediate host snail is around one year and each year, in the early transmission season (May/June), snails infected during the previous transmission season contaminate the water-bodies. During the flooding that starts in July, most of this infected older snail generation die off, while a new generation becomes infected. The sporocyst schistosome stage in these newly infected snails mature in August and the snails start shedding cercariae in September. The June peak was never remarkable compared to the September one since the routinely applied large-scale, spring mollusciciding programme regularly reduces the number of previously infected snails, while the new generation is produced later in the year and is therefore less affected. Consequently, the emergence of new, infective snails between August and October along the river puts the area at risk for schistosomiasis infection and emphasizes the importance of applying control measures at this time of the year as well as in the spring. This was done in 2010, which resulted in only one small peak at the early surveillance stage that year and led to the dramatic reduction in risk in spatiotemporal terms (number of positive sites and months at risk) compared to that of the previous year.

## Conclusions

In conclusion, the improved situation was attributed to the following reasons: 1) reinforced mollusciciding control strategy at the hotspots in the spring as well as in the autumn of 2010; 2) health control propaganda in August 2010 with emphasis on the mobile population working on the Yangtze River attempting to reduce contamination of the water with schistosome eggs; and 3) screening and treatment of target populations.

We found the most dangerous environments for schistosome infection to be the river ports, ferry terminals and public vessel centres with human activities. Enhanced prevention and control efforts should target these areas. Interestingly, two surveillance sites with positive sentinel mice had no snails or a very low density of snails and this situation demands further investigation. Another unknown question is the source of miracidia infecting the snails during flooding. During this season, most of the marshlands of the Yangtze River are submerged making it impossible for livestock to graze on the beaches. It is hypothesized that the miracidia may come from the exogenous faeces discharged by transportation boats in the Yangtze River. However, this also needs to be confirmed by further study.

People may raise a critical point that this system may not be able to get real-time infection information since the earliest detection time of the positive results is about 35-40 days after the sentinel mice are put into the field. However, on the other hand, it allowed us to carry on more targeted prevention and control of schistosomiasis along the Yangtze River, which means the control mode gradually changed from conventional large-scale approach to pinpoint control. This monitoring system made the implementation of schistosomiasis control more precise, which in turn further improved the cost-effectiveness of the conventional control process. Thus, small control inputs can generate great control effect. The future requires multivariate spatial analysis for the production of reliable risk maps showing high-risk areas and the scope of distribution. This should further guide the on-site emergency control work [19].

## Competing interests

The authors declare that they have no competing interests.

## Authors' contributions

SLP, LYS, GJY and ZXN conceived the study. GJY wrote the first version of the manuscript. All authors helped in the field experiments in Jiangsu provinces. XNZ revised the manuscript. All of authors read, contributed to, and approved the final version of the manuscript.
